# Spatially Analyzing the Inequity of the Hong Kong Urban Heat Island by Socio-Demographic Characteristics

**DOI:** 10.3390/ijerph13030317

**Published:** 2016-03-12

**Authors:** Man Sing Wong, Fen Peng, Bin Zou, Wen Zhong Shi, Gaines J. Wilson

**Affiliations:** 1 Department of Land Surveying and Geo-Informatics, The Hong Kong Polytechnic University, Kowloon, Hong Kong, China; lswong@polyu.edu.hk (M.S.W.); fen.peng@connect.polyu.hk (F.P.); john.wz.shi@polyu.edu.hk (W.Z.S.); 2 Joint Spatial Information Research Laboratory between The Hong Kong Polytechnic University and Wuhan University, Wuhan 430072, China; 3 School of Geosciences and Info-Physics, Central South University, Changsha 410083, China; 4 Department of Biological Sciences, Huston-Tillotson University, Austin, TX 78702, USA; jwilson@htu.edu

**Keywords:** environmental inequity, land surface temperature, socio-demographic characteristic, spatial autocorrelation, urban heat island

## Abstract

Recent studies have suggested that some disadvantaged socio-demographic groups face serious environmental-related inequities in Hong Kong due to the rising ambient urban temperatures. Identifying heat-vulnerable groups and locating areas of Surface Urban Heat Island (SUHI) inequities is thus important for prioritizing interventions to mitigate death/illness rates from heat. This study addresses this problem by integrating methods of remote sensing retrieval, logistic regression modelling, and spatial autocorrelation. In this process, the SUHI effect was first estimated from the Land Surface Temperature (LST) derived from a Landsat image. With the scale assimilated to the SUHI and socio-demographic data, a logistic regression model was consequently adopted to ascertain their relationships based on Hong Kong Tertiary Planning Units (TPUs). Lastly, inequity “hotspots” were derived using spatial autocorrelation methods. Results show that disadvantaged socio-demographic groups were significantly more prone to be exposed to an intense SUHI effect: over half of 287 TPUs characterized by age groups of 60+ years, secondary and matriculation education attainment, widowed, divorced and separated, low and middle incomes, and certain occupation groups of workers, have significant Odds Ratios (ORs) larger than 1.2. It can be concluded that a clustering analysis stratified by age, income, educational attainment, marital status, and occupation is an effective way to detect the inequity hotspots of SUHI exposure. Additionally, inequities explored using income, marital status and occupation factors were more significant than the age and educational attainment in these areas. The derived maps and model can be further analyzed in urban/city planning, in order to mitigate the physical and social causes of the SUHI effect.

## 1. Introduction

Environmental inequity can be defined as a type of inequality when a particular social group is disproportionately burdened with environmental problems, e.g., air pollution and heat stress [1,2,3]. The underlying contributors to environmental inequity can be political, economic, and social factors [4,5]. Hong Kong is a highly urbanized and densely populated city with a complex and dense population spatial distribution, including different ages, educational attainments, occupations, marital statuses, and incomes. Hong Kong also has one of the highest income inequities in the world [6]. The Urban Heat Island (UHI) is a phenomenon in which ambient air temperature in the city center is elevated relative to surrounding non-urbanized areas [7,8]. The main causes of UHI include: (i) the replacements of soil and vegetation by impervious surfaces, e.g., concrete and asphalt; (ii) urban structures, e.g., tall buildings and narrow streets; and (iii) anthropogenic heat discharges [9]. As a consequence of rising temperature, the numbers of people suffering adverse heat health effects (e.g., heat exhaustion, heat stroke) are expected to increase, and these effects may be disproportionately distributed over disadvantaged populations [10]. UHI is strongly related to the built environment [11,12,13] as well as some socioeconomic and demographic characteristics [14,15,16]. Huang *et al.* found that the land surface temperatures were highly correlated with poverty, lower education, ethnic minorities, and elderly people in Baltimore, U.S. [15]. In Hong Kong, Chan *et al.* found that people younger than 75 years, married, and living in districts of low socioeconomic status were more susceptible to high temperatures, which lead to the higher of mortality rates among these groups [16].

There are several methods used to measure the UHI: (i) traditional observations based on atmospheric observation data from monitoring stations [17]; (ii) remote sensing techniques that derive land surface and air temperature [18], vegetation indices [19], and thermal landscape [20]; and (iii) modelling and prediction, e.g., boundary layer models [21,22]; (iv) fixed-point observation method; and (v) transect sampling method. Due to the sparse and unevenly distribution of monitoring stations in Hong Kong, remote sensing data thus provide a synoptic observation covering the entire territories. A Surface Urban Heat Island (SUHI) can be defined as the UHI effect measured by land surface temperature. With the results of the SUHI, an inequity analysis of population exposure to the SUHI for specific socio-demographic groups can therefore be conducted [14,15,23].

This study aims to: (i) investigate whether any groups of socio-demographic characteristics or disadvantaged groups are more likely to reside in urban heat island core areas in Hong Kong; and (ii) examine the spatial distribution patterns of the inequity hotspots in Hong Kong. The study results can provide crucial information to help decision-makers in Hong Kong and other similar administrations understanding the spatial inequities of environmental burdens over geographical area, and thus prioritize interventions to mitigate death/illness rates from heat for disadvantaged populations.

## 2. Materials and Methods

### 2.1. Study Area

**Figure 1:**
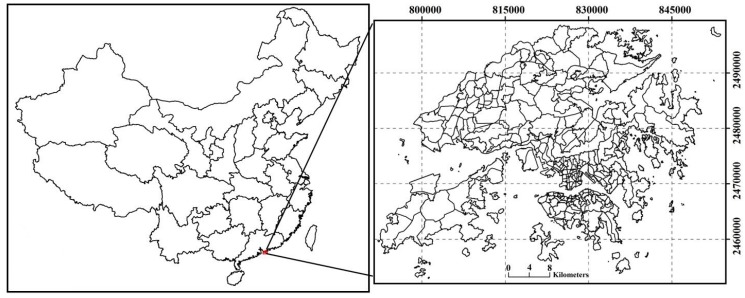
Study area of Hong Kong. **Left**: province boundary of China; **Right**: Tertiary Planning Units (TPU) of Hong Kong.

Hong Kong is located at latitude 22°9′14″ N~22°33′44″ N, longitude 113°50′7″ E~114°26′30″ E (Figure 1), and is one of two special administrative regions of the People’s Republic of China. It is situated on the south-eastern coast of mainland China, with a total population of 7.19 million in 2013, which consists of Hong Kong Island (17.7% of the total population), Kowloon Peninsula (30.0% of the total population), and New Territories (52.2% of the total population) [24]. Hong Kong is situated in a humid sub-tropical climate, where spring is warm and wet, and autumn is warm and dry. Approximately 1948 h of sunshine can be observed per year, and about 90% of the rainfall occurs between April and September [25]. Monthly mean air temperatures range between 16.1 °C and 28.7 °C. The record high and low temperatures ever recorded by the Hong Kong Observatory (HKO) are, respectively, 36.1 °C on 19 August 1900 and 18 August 1990, and 0.0 °C on 18 January 1893 [26]. The mean monthly air temperature on November 2005 was 23.0 °C, based on data observed at the Hong Kong International Airport.

### 2.2. Data Sources and Analysis

Memon *et al.* [27] stated that the maximum values of mean monthly UHI (urban-rural temperature) in the year 2005 were observed in winter and spring, and the second highest UHI values were also observed in autumn in Hong Kong. Thus, a cloud-free Landsat 5 Thematic Mapper (TM) image on23 November 2005 was used in this study. The day of 23 November 2005 was cloud-free and with low wind speed, and there was no rainfall within +/− 3 days during the data acquisition. The Landsat 5 TM image was orthorectified to the World Geodetic System (WGS) 84 Universal Transverse Mercator (UTM) zone 49N coordinate system.

In addition, Hong Kong topographic and digital maps were acquired and used in the study. The 2006 population data at Tertiary Planning Units (TPU) in Hong Kong were retrieved from the Census and Statistics Department of Hong Kong [28], which provides open data sets [29]. The boundaries of TPUs are demarcated by the Planning Department of the Government of the Hong Kong Special Administrative Region (HKSAR). TPUs typically represent geographical areas bounded by roads, railway lines, coastlines, contours, waterways, lot boundaries or zoning boundaries of town plans, which can provide a common geographic system for the compilation of statistical data [30]. The boundaries of TPUs are regularly updated to reflect population dynamics, in which a TPU of less than 1000 persons is merged with an adjacent TPU. A TPU is a geographic unit used by the Government for planning purposes. The entire territory of Hong Kong was divided into 287 TPUs in 2006.

### 2.3. Methodology

A suite of methods including remote sensing retrieval algorithms, Population Dynamic Mapping Models (PDMM), logistic regression models and spatial autocorrelation analysis in Geographical Information Systems (GIS) were utilized to evaluate inequities in exposure to the SUHI in Hong Kong.

#### 2.3.1. Derivation of Land Surface Temperature Image

This study applied a mono-window algorithm to retrieve the Land Surface Temperature (LST) from a 2005 Landsat TM image at 30 m spatial resolution. A mono-window algorithm is appropriate for retrieval using only a single thermal band, whereas the split-window algorithm is recommended for two thermal channels [31]. The land surface temperatures were divided into six classes (e.g., low, sub-low, medium, sub-high, high, and extreme high temperature) based on the mean and standard deviation (SD) of the temperature distribution [32], in which the classes of high and extreme high temperature were identified as the SUHI core areas. The details on the LST retrieval are available from previous studies [33]. The rationale for categorizing SUHI or non-SUHI areas of a 120 m × 120 m grid is to find whether the total grid numbers of 30 m × 30 m pixels with high and extreme high temperatures are greater than eight (half of the total pixel number) inside the 120 m grid.

#### 2.3.2. Reclassification of Socio-Demographical Indicators and LST

Following previous studies [4,34,35], groupings of age, income, educational attainment, marital status and occupation were deemed to be valid representations of socio-demographic indicators. These characteristics were categorized into different levels based on the reference categories (Table 1) [4]. In order to characterize SUHI exposure inequity using the logistic regression model, SUHI areas should first be identified and validated. The thresholds and classes of LST were determined using Equation (1):

T = µ ± χ·SD
(1)

where T denotes temperature threshold value, μ is mean land surface temperature of Hong Kong, SD is standard deviation of LST, and χ denotes multiple of deviation (values is assigned 0.5, 1.0) [36]. Table 2 displays the derived temperature ranges and classes of land surface temperature over Hong Kong.

#### 2.3.3. Population Density by Socio-Demographic Characteristics

The population density in Hong Kong was modelled using the PDMM method. The socio-demographic characteristic data were presented by TPU in different homogenous zones based on a combination of areal weighting and an urban-classification method [37]. Empirical sampling provides a proportional density fraction used as a weighted value representing each urban class. The urban class is based upon land use and land cover data at 30 m spatial resolution. The ArcMap extension module “dasymetric mapping” was used to model population density, which can automate the areal interpolation process within a GIS framework [38]. The modelling of population distribution using PDMM at relatively high spatial resolution data has been used in previous studies [39,40]. Since the populations of 30 m × 30 m grids in some sparsely populated areas were almost close to zero, the lower resolution 120 m × 120 m grid was thus selected to calculate the ORs of the TPUs, where there are 16 grids in order to ensure data quality.

#### 2.3.4. Logistic Regression Model

In terms of the nominal variable (SUHI/non-SUHI areas) and socio-demographic variable at 120 m × 120 m grids (*i.e.*, which indicates that each TPU has dozens of grids for regression modeling), a binary logistic regression was adopted to calculate the OR value of each TPU at the 120 m spatial resolution using the SPSS software package (International Business Machines Corporation (IBM), New York, NY, USA). In this process, the nominal variable classified as SUHI areas and non-SUHI areas was the dependent variable (within the SUHI core areas were coded as 1, and outside the SUHI core areas were coded as 0). Socio-demographic factors were the independent variable (the reference and target groups were coded as 0 and 1, respectively). The population numbers with different socio-demographic characteristics at 120 m × 120 m grids were input as weights in the calculation. Thus, a total of 287 Odds Ratio (OR) values were calculated in Hong Kong as the number of TPUs by each categorical socio-demographic factor. The Odds Ratio value indicates the relative amount by which the odds of the outcome increases (OR greater than 1.0) or decreases (OR less than 1.0) as the predictor value increases by 1.0 unit.

#### 2.3.5. Spatial Autocorrelation Method

(i) Global Autocorrelation Analysis

Global spatial analysis or global spatial autocorrelation analysis yields only one statistical result to summarize the entire study area. In short, global spatial analysis assumes homogeneity within the study area. In practice, the homogeneity assumption may not hold. Thus having only one statistical result may not be suitable for diverse and more complex spatial patterns [41,42].

There are several ways to test the global autocorrelations of events. The most popular among spatial autocorrelation methods is Moran’s I statistic, which is used to test the null hypothesis that the spatial autocorrelation of a variable is zero [43,44]. If the null hypothesis is rejected, the variable will be considered spatially autocorrelated. Moran’s I statistic of spatial autocorrelation is described by Cliff and Ord [45].

In this study, the Hong Kong TPU was used as the base spatial unit [46]. Spatial autocorrelation analysis was developed based on the ORs value of TPUs by categorical socio-demographic factors. Moran’s I index indicates the extent of global spatial autocorrelations of SUHI inequities by categorical socio-demographic indicators (*i.e.*, age, income, educational attainment, marital status, and occupation). The analyses were conducted using the “Univariate Moran’s I” in GeoDa.

(ii) Local Hotspot Detection

Although global autocorrelation has been observed, the more detailed local pattern of hot spots requires further assessment. The challenge is in finding an appropriate test for local spatial autocorrelation in the presence of global spatial autocorrelation. Univariate local Moran’s I-based cluster mapping has been suggested as an effective method in detecting the hot spots or cluster areas of environmental exposure inequity based on spatial autocorrelation theory [45]. To identify hot spots or cluster areas that are statistically significant, the cluster and outlier analysis [47] functions were utilized to identify local spatial patterns of the SUHI inequity in the study area.

Moran’s I index calculates the difference between the target and the mean for all values, with a range between −1.0 and +1.0 [47]. If spatial objects that are located more closely together have similar attributes (*i.e.*, high values near high values; low values near low values), Moran’s I index will be positive, and if different attributes are located close together, the index value will be negative. If the values in the dataset tend to be stochastic spatially, the index will be near zero.

The Univariate local Moran’s I in GeoDa was utilized to identify local hot spot areas of SUHI exposure inequity in this study [48]. A z-score is used to test the null-hypothesis. A high positive z-score (z > 3.29) for SUHI exposure inequities of a TPU with *p* value at the 99.9% confidence level indicates the surrounding features have either high or low OR values (*i.e.*, high-high, or low-low). Inversely, a low negative z-score (z < −3.29) for SUHI exposure inequities in a TPU with *p* value at the 99.9% confidence level indicates a significant spatial outlier (*i.e.*, high-low, or low-high) [49]. High-high areas are deemed to high-risk areas of spatial inequities by socio-demographic characteristics in the study.

Currently, there are two basic categories of neighbor definitions: contiguity (shared borders) and distance. Contiguity-based weights matrices include rook and queen. Distance-based weights matrices include distance bands and k nearest neighbors [50]. For accurate detection of the hotspot areas with SUHI exposure inequity in this study, “k-nearest neighbors” belonging to “Distance-based weights matrices” were employed to create spatial weights. This is because the size of TPU areas varies considerably and some of them do not share borders with neighbors. In this process, the number of k-nearest neighbors was set as six in order to ensure that every polygon has the same number of neighbors.

## 3. Results

### 3.1. Distribution of Urban Heat Islands in Hong Kong

**Figure 2:**
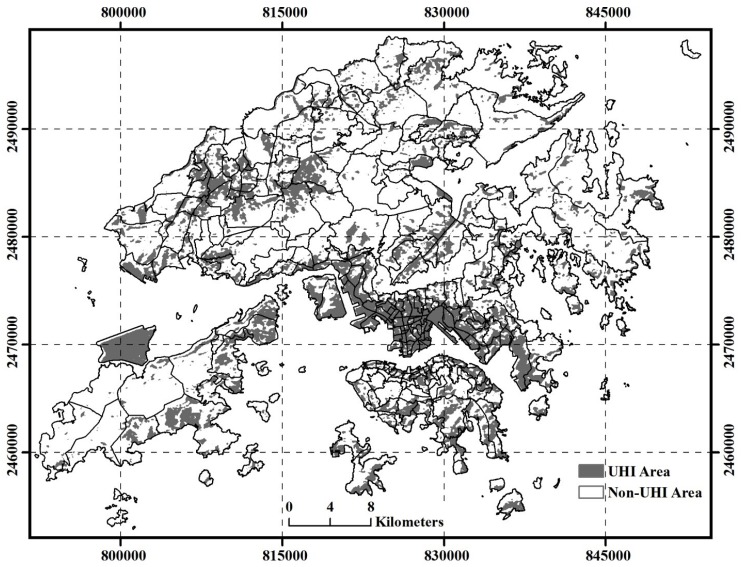
Spatial distribution of Surface Urban Heat Island (SUHI) areas in Hong Kong. The SUHI map was derived based on temperature ranges and classes of land surface temperature, in which the classes of high and extreme high temperatures were identified as the SUHI core areas.

For the Landsat TM data, the mean temperature of the LST was 24.8 °C. Figure 2 indicates there is an observable and strong urban heat island effect in Hong Kong. These SUHI core areas are mainly distributed in several highly urbanized areas such as the commercial centers in Kowloon Peninsula, the northern Hong Kong Island and Hong Kong International Airport.

### 3.2. Inequity of SUHI by Socio-Demographic Characteristics

#### 3.2.1. Global Autocorrelation Analysis of SUHI Inequity

Table 3 displays the frequency of ORs which are greater than 1, by socio-demographic characteristics at the TPU level, where the proportion is the number of TPUs with ORs greater than 1, divided by the total number of TPUs in Hong Kong. From Table 3, it can be seen that the age group of 60+ years was more likely to be exposed to SUHI areas in some TPUs. The age group of 60+ years had a higher proportion (64.9%) than age group 0 to 14 (33.3%), where the inequity of age group 0 to 14 was not significant, although the mean OR value is larger than 1. Income groups less than HK$4000 per month, HK$4000 to HK$10,000, HK$10,000 to HK$20,000, HK$20,000 to HK$40,000 were more likely to be exposed to the SUHI core areas, however, income group HK$20,000 to HK$40,000 per month had the highest proportion (66.7%). The groups with educational attainment of secondary and matriculation were more likely to be exposed to the SUHI core areas. Additionally, secondary and matriculation educational level have a higher proportion (64.9%) than pre-primary and primary groups (33.3%). Widowed, divorced and separated people are more likely to be exposed to the SUHI core areas. Widowed, divorced and separated people have a higher proportion (66.7%) than unmarried people (53.3%). All occupational groups including clerks, service workers, shop sales workers, craft and related workers, plant and machine operators, assemblers, and elementary occupations, skilled agricultural and fishery workers, and occupations not classified were also more likely to be exposed to the SUHI core areas. Clerks, service workers, shop sales workers had the highest proportion (63.0%), following by craft and related workers, plant and machine operators, assemblers (60.2%), and elementary occupations, skilled agricultural and fishery workers, and occupations not classified (52.9%).

Table 4 shows the values calculated by Global autocorrelation for Hong Kong. From the Univariate Moran’s I index, pre-primary and primary educational attainment groups showed statistically significant clusters, and had the largest positive index values (0.958). All groups showed significant spatial cluster patterns, except for the age groups of 60+ years, unmarried people, and craft and related workers, plant and machine operators. Assemblers were not statistically significant.

#### 3.2.2. Spatial Clustering of SUHI Inequity by Age

**Figure 3:**
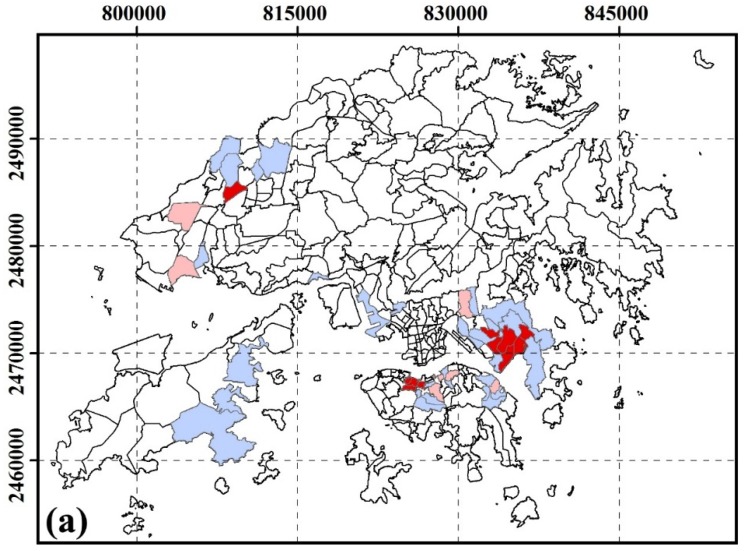

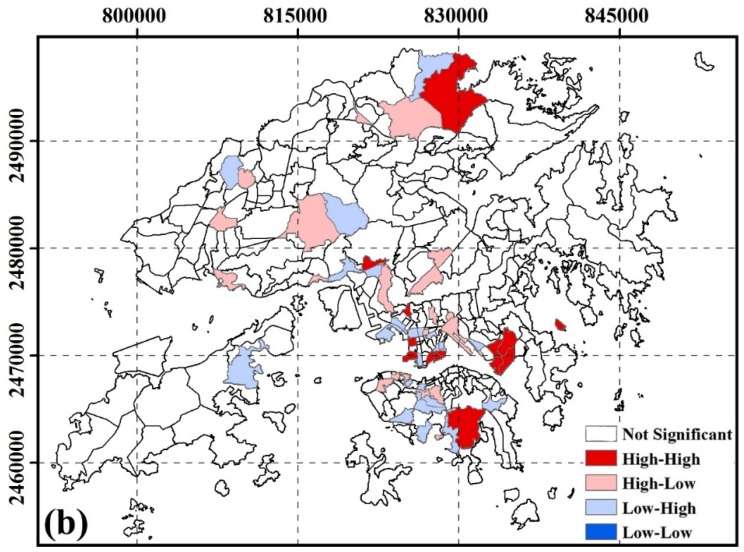
Spatial cluster residing in SUHI core area by age at TPU level. (**a**) age < 14; (**b**) age > 60. A local autocorrelation method was used to identify statistically significant hot spots. High-High areas indicate high OR values near high OR values; Low-Low areas indicate low OR values near low OR values; High-Low areas indicate high OR values near low OR values; Low-High areas indicate low OR values near high OR values.

Figure 3 shows the inequity of SUHI exposure by age groups. High-risk areas for age groups of less than 14 years were located in Central District, Admiralty, Queen’s Road East, Mid-level, Sau Mau Ping, Lam tin, Shek Po Tsuen, Chiu Keng Wan Shan, Ng Kwai Shan, Hang Hau Junk Bay. High-risk areas for age groups of more than 60 years were located in Cheung Lin Shan, Hung Hom, Hong Kong Coliseum, The Arch, Tai Kok Tsui, So Uk, Lam tin, Yau Tong, Lo Wai, Sha Tau Kok, Tai Po Tsai, Chiu Keng Wan Shan, Ng Kwai Shan.

#### 3.2.3. Spatial Clustering of SUHI Inequity by Income

**Figure 4:**
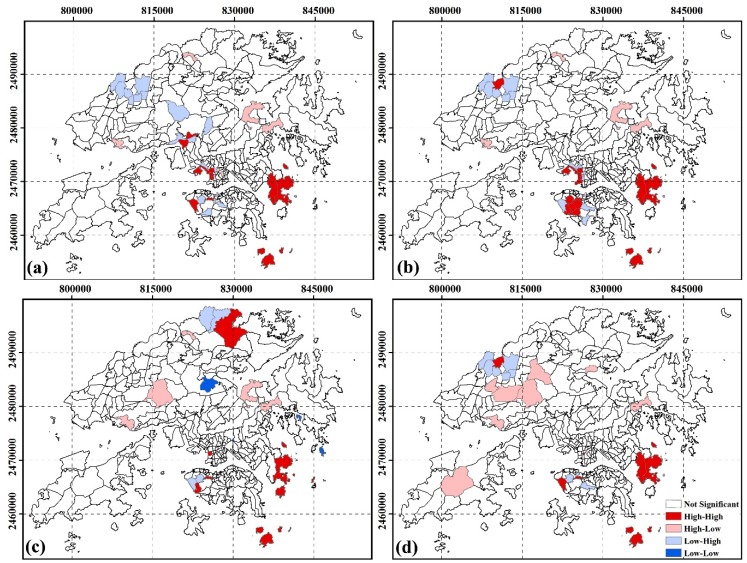
Spatial cluster residing in SUHI core area by income at TPU level. (**a**) income < $4000; (**b**) income from $4000 to $10,000; (**c**) income from $10,000 to $20,000; (**d**) income from $20,000 to $40,000.

Figure 4 shows the inequity of SUHI exposure by income. High-risk areas for incomes less than HK$4000 per month were located in Mid-level, Pok Fu Lam, Shek O, Mong Kok, Yau Ma Tei, Tai Kok Tsui, Ngong Shuen Chau, Lo Wai, Tsuen Wan, Tai Po Tsai, Clear Water Bay, Siu Chik Sha, Po Toi Islands. High-risk areas for incomes between HK$4000 and HK$10,000 per month were located in Mid-level, Wah Fu, Aberdeen, The Peak, Mong Kok, The Arch, Yau Ma Tei, Tai Kok Tsui, Ngong Shuen Chau, Sheung Shui, Tai Po Tsai, Clear Water Bay, Siu Chik Sha, Po Toi Islands. High-risk areas for incomes between HK$10,000 and HK$20,000 per month were located in Mid-level, Wah Fu, Tai Kok Tsui, Sha Tau Kok, Tai Po Tsai, Clear Water Bay, Tung Lung Chau, Po Toi Islands. High-risk areas for incomes between HK$20,000 and HK$40,000 per month were located in Mid-level, Pok Fu Lam, Sheung Shui, Tai Po Tsai, Clear Water Bay, Siu Chik Sha, Po Toi Islands.

#### 3.2.4. Spatial Clustering of SUHI Inequity by Marital Status

**Figure 5:**
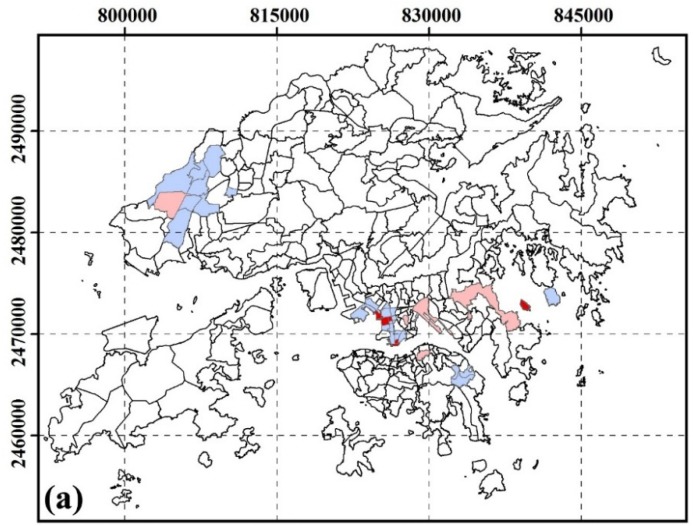

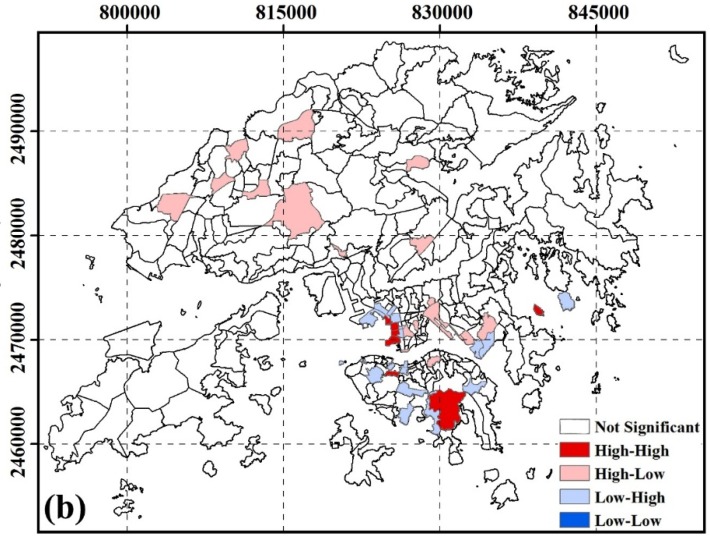
Spatial cluster residing in SUHI core area by marital status at TPU level. (**a**) unmarried; (**b**) widowed and divorced and separated.

Figure 5 shows the inequity of SUHI exposure by marital status. High-risk areas for unmarried groups were located in East Tsim Sha Tsui, Tsim Sha Tsui Ferry Pier, Mongkok South, Tai Kok Tsui, Tai Po Tsai. High-risk areas for widowed, divorced and separated groups were located in Mid-level, Cheung Lin Shan, The Arch, Yau Ma Tei, Tai Kok Tsui, Tai Po Tsai.

#### 3.2.5. Spatial Clustering of SUHI Inequity by Occupation

**Figure 6:**
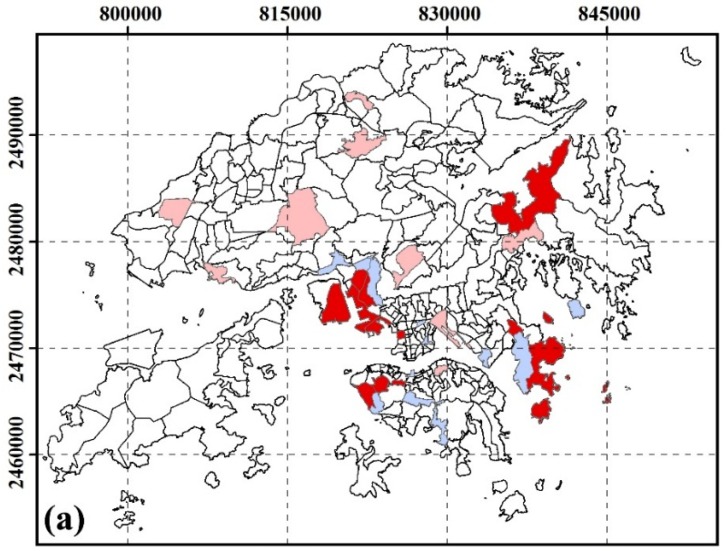

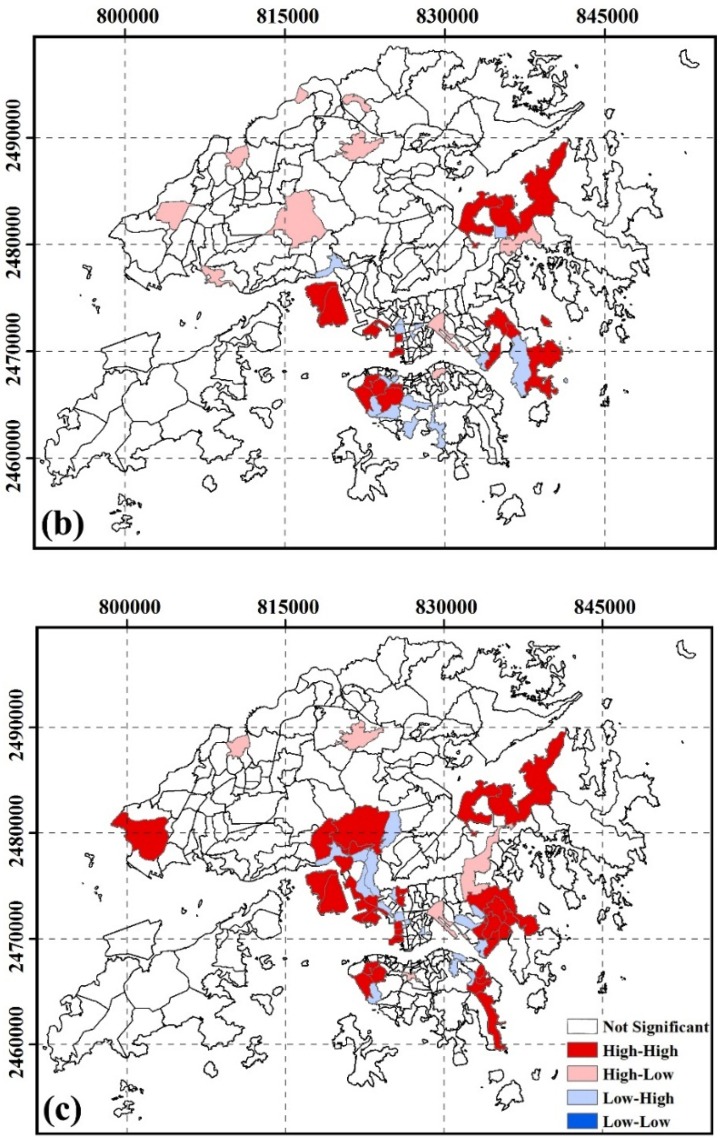
Spatial cluster residing in SUHI core area by occupation at TPU level. (**a**) clerks, service workers, shop sales workers; (**b**) craft and related workers, plant and machine operators, assemblers; (**c**) elementary occupations, skilled agricultural and fishery workers, occupations not classified.

Figure 6 shows the inequity of SUHI exposure by occupation. High-risk areas for clerks, service workers, shop sales workers were located in Mid-level, Pok Fu Lam, The Peak, Shek O, Tai Kok Tsui, Ngong Shuen Chau, Container Terminal, Kwai Shing Estate, Ha Kwai Chung, Tsing Yi, Kei Ling Ha Hoi, Tai Po Tsai, Clear Water Bay, Tung Lung Chau, Hang Hau. High-risk areas for craft and related workers, plant and machine operators, and assembler occupation groups were located in Hong Kong West, Mid-level, Pok Fu Lam, The Peak, The Arch, Tai Kok Tsui, Ngong Shuen Chau, Tsing Yi, Shek Wan, Kei Ling Ha Hoi, Ma On Shan, Tai Po Tsai, Clear Water Bay, Chiu Keng Wan Shan, Po Lam, Hang Hau. High-risk areas for elementary occupations, skilled agricultural and fishery workers, and occupations not classified were located in Hong Kong West, Chai Wan Au, Tsui Lok Estate, Chai Wan, Pok Fu Lam, The Peak, Shek O, The Arch, Yau Ma Tei, Tai Kok Tsui, Lai chi Kok, Shek Kip Mei, Ngong Shuen Chau, Lam tin, Tai Mo Shan, Container Terminal, Lo Wai, Tso Kung Tam, Tsuen Wan, Sheung Fa Shan, Tsing Yi, Shek Wan, Tai Lang Shui, Kei Ling Ha Hoi, Ma On Shan, Tai Po Tsai, Chiu Keng Wan Shan, Ng Kwai Shan, Po Lam, Hang Hau, Junk Bay.

#### 3.2.6. Spatial Clustering of SUHI Inequity by Educational Attainment

**Figure 7:**
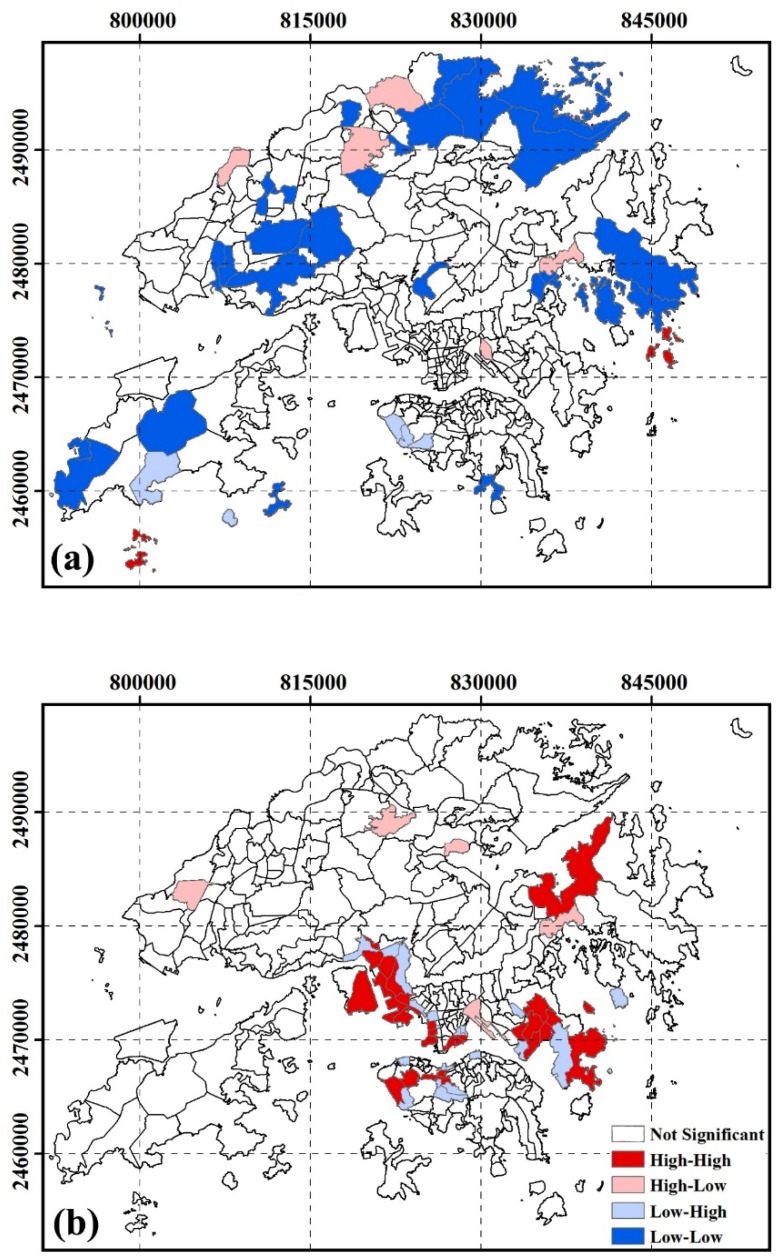
Spatial cluster residing in SUHI core area by educational attainment at TPU level. (**a**) pre-primary and primary; (**b**) secondary and matriculations.

Figure 7 shows the inequity of SUHI exposure by educational attainment. High-risk areas for pre-primary and primary groups were located in Basalt Island, Town Island, Clear Water Bay, Soko Island. High-risk areas for secondary and matriculation groups were located in Queen’s Road East, Wan Chai, Mid-level, Pok Fu Lam, The Peak, Hung Hom, East Tsim Sha Tsui, Hong Kong Coliseum, The Arch, Yau Ma Tei, Tai Kok Tsui, Lai chi Kok, Ngong Shuen Chau, Lam tin, Container Terminal, Tso Kung Tam, Tsuen Wan, Kwai Shing Estate, Ha Kwai Chung, Tsing Yi, Kei Ling Ha Hoi, Clear Water Bay, Chiu Keng Wan Shan, Ng Kwai Shan, Po Lam, Hang Hau, Junk Bay.

## 4. Discussion

This study investigated whether socio-demographic characteristics of a TPU are indicative of the exposure to excessive heat combining thermal satellite image and census data. The urban heat island core areas co-occurred at the TPU scale with low socio-demographic characteristics as measured by vulnerable age, secondary and matriculation education, widowed, divorced and separated people. All income groups of less than $40,000 and occupation groups including clerks, service workers, shop sales workers, craft and related workers, plant and machine operators, assemblers, and elementary occupations, skilled agricultural and fishery workers, and occupations not classified showed similar exposure to SUHI core areas. Chan *et al.* [16] stated that low socio-economic status groups were more sensitive than other groups to high temperature effects in Hong Kong and other previous studies investigated how many socially vulnerable hotspot areas were located in the LST hotspot areas. However, our study highlighted the spatial clusters of SUHI inequity by age, income, educational attainment, marital status, and occupation within the TPU spatial units. Our study extended the findings from previous research by incorporating the spatial perspective of these inequities.

Although this study has revealed the spatial cluster of some disadvantaged groups and some socially vulnerable hotspots areas in Hong Kong, some limitations of both data and techniques persist as follows: (i) this study used the aggregate census data in TPU spatial units. However, data of individual-level information such as individual activities due to work, recreation and living are not available. Compared to previous studies using census units (e.g., census tract, block group), this study used finer geographical units with the aid of PDMM, which considers land use type for population distribution. The appropriate resolution/unit with respect to the areal coverage and data aggregation issue in the analysis of spatial inequity will be further studied in future research; (ii) according to the results of spatial autocorrelation, some vulnerable areas are in remote areas, and this might be possibly attributed to a combination between diversities of land use types resulting in spatial discrepancy between the SUHI core areas (*i.e.*, SUHI core areas and non-core areas present in a TPU) and the differences in population distribution. Although it seems that the proportions of mean population in the SUHI core areas *versus* non-SUHI core areas in target groups (e.g., age groups of less than 14 years) are greater than the proportions of mean population in SUHI core areas *versus* non-SUHI core areas in reference group (e.g., ages between 14 and 60 years old). A testing of causal hypotheses through analyzing different factors caused by SUHI and population distribution may explain these abnormal distributions; (iii) it is difficult to eliminate the potential bias of the logistic regression modelling for each type of demographic variable (e.g., age) by inputting the remaining variables (e.g., education attainment, income) as confounding factors, since the attribute values for those variables are aggregated values rather than at the individual level; (iv) due to data availability, the LST data used in this study were derived from a daytime image. The nighttime temperature difference between urban and rural areas will usually be more significant than the daytime, but a nighttime image covering the entire area of Hong Kong was not available. More detailed study of using a time-series of Landsat and HJ-1A/1B thermal satellite images and years of population and census data to analyze a time-series of spatial inequality will be conducted in future research. In addition, LST data will be studied and used to derive air temperature image covering the territories. More comprehensive datasets including urban morphological and dynamic traffic datasets will be coupled in the future study.

It should be noted that this study is fairly novel in the methodology employed (e.g., spatial autocorrelation) for investigating environmental and socio-demographic inequities (geographic unit, PDMM, statistical analysis methods, definition of social deprivation). Therefore, the results provided in this study would be highly applicable in other study disciplines and areas.

## 5. Conclusions

In conclusion, a spatial autocorrelation method was used to analyze the relationships between socio-demographic variables and spatial patterns of SUHI exposure. Based on an LST image from 2005, the study revealed that the populations likely to be exposed to intense SUHI areas are comprised largely of disadvantaged socio-demographic groups, including age groups of 60+ years, secondary and matriculation educational level, widowed, divorced and separated people, low and middle income groups, and occupation groups of workers in Hong Kong. This study also indicated spatial patterns of inequity hotspots in Hong Kong. The results of this study and methods used herein may provide valuable decision-making information to government, planning authorities, and various other stakeholders, who can respond to mitigate potential inequities for disadvantaged communities, as well as to prioritize for heat prevention and intervention works. The study can also be extended for future city planning, such as tree-planting, to help reduce heat exposure and mitigate the urban heat island effect. Policy-makers can also adapt the urban infrastructure and community services to assist vulnerable groups in SUHI exposure.

## Figures and Tables

**Table 1:** Standards of categorization and reference categories for socio-demographic measurements.

Age	Income (HK$ per Month)	Educational Attainment	Marital Status	Occupation
0–14	<4 K	Pre-primary Primary	Unmarried	Managers, Administrators, Professionals, Associate Professionals ^1^
14–60 ^1^	4 K–10 K	Secondary Sixth form	Married ^1^	Clerks, Service Workers, Shop Sales Workers
>60	10 K–20 K	Post-secondary ^1^	Widowed Divorced Separated	Craft and Related Workers, Plant and Machine Operators, Assemblers
	20 K–40 K			Elementary Occupations, Skilled Agricultural and Fishery Workers, Occupations not classified
	>40 K ^1^			

^1^ Reference category for comparison based on existing studies.

**Table 2:** Temperature ranges and classes of land surface temperature in Hong Kong.

Temperature Range	Class
Extreme high temperature area	TS > μ+ SD
High temperature area	μ + 0.5SD < Ts ≤ μ + SD
Sub-high temperature area	μ < Ts ≤ µ + 0.5SD
Medium temperature area	μ − 0.5SD ≤ Ts ≤ μ
Sub-low temperature area	μ – SD ≤ Ts<μ − 0.5SD
Low temperature area	Ts < μ − SD

**Table 3:** Frequency of Odds Ratios (ORs) greater than 1 by socio-demographic characteristics.

Characteristics	Level	Count (%) ^1^	Minimum (95% CI)	Maximum (95% CI)	Mean
Age	<14	11(33.3%)	1.04(1.00,1.08)	1.39(1.20,1.61)	1.12
	>60	50(64.9%)	1.03(1.00,1.06)	2.58(1.37,4.85)	1.33
Income (HK$ per month)	<4 K	56(57.7%)	1.07(1.00,1.14)	6.11(2.16,17.28)	1.70
	4 K–10 K	67(60.9%)	1.09(1.00,1.19)	7.25(4.49,11.70)	2.01
	10 K–20 K	61(61.0%)	1.07(1.00,1.15)	8.61(1.14,65.05)	2.02
	20 K–40 K	44(66.7%)	1.11(1.02,1.21)	7.13(2.48,20.51)	1.75
Educational attainment	Pre-primary Primary	61(47.3%)	1.05(1.02,1.08)	7.68(5.89,10.01)	1.89
	Secondary Sixth form	56(60.9%)	1.03(1.00,1.06)	3.75(2.55,5.50)	1.36
Marital status	Unmarried	8(53.3%)	1.07(1.02,1.12)	1.20(1.11,1.30)	1.13
	Widowed Divorced Separated	28(66.7%)	1.07(1.01,1.13)	1.95(1.60,2.38)	1.34
Occupation	Clerks, Service Workers, Shop Sales Workers	51(63.0%)	1.04(1.00,1.07)	4.26(3.06,5.93)	1.47
	Craft and Related Workers, Plant and Machine Operators, Assemblers	53(60.2%)	1.06(1.02,1.10)	8.84(5.75,13.59)	1.82
	Elementary Occupations, Skilled Agricultural and Fishery Workers, Occupations not classified	46(52.9%)	1.04(1.00,1.07)	1.95(1.78,2.13)	1.24

^1^ The proportion is the number of TPUs with ORs greater than 1, divided by the total number of TPUs in Hong Kong.

**Table 4:** Univariate Moran’s I statistics by age, income, educational attainment, marital status, and occupation.

Characteristics	Level	Moran’s I	z-Value
Age	<14	0.226	9.596
	>60	0.176	7.492
Income (HK$ per month)	<4 K	0.393	16.138
	4 K–10 K	0.347	14.708
	10 K–20 K	0.565	24.118
	20 K–40 K	0.455	18.435
Educational attainment	Pre-primary Primary	0.801	34.565
	Secondary Sixth form	0.360	14.471
Marital status	Unmarried	0.139	6.088
	Widowed Divorced Separated	0.101	4.360
Occupation	Clerks, Service Workers, Shop Sales Workers	0.490	20.549
	Craft and Related Workers, Plant and Machine Operators, Assemblers	0.276	12.303
	Elementary Occupations, Skilled Agricultural and Fishery Workers, Occupations not classified	0.260	10.955

## Abbreviations

The following abbreviations are used in this manuscript:

SUHI	Urban Heat Island
TM	Thematic Mapper
LST	Land Surface Temperature
PDMM	Population Dynamic Mapping Model
TPU	Tertiary Planning Units
ORs	Odds Ratios
OLS	Ordinary Least Squares Models
GIS	Geographical Information System
LULC	land use and land cover
HKO	Hong Kong Observatory
HKSAR	Hong Kong Special Administrative Region
